# Clinical Implications of Adipocytokines and Newly Emerging Metabolic Factors with Relation to Insulin Resistance and Cardiovascular Health

**DOI:** 10.3389/fendo.2013.00097

**Published:** 2013-08-21

**Authors:** Sung Hee Choi, Eun Shil Hong, Soo Lim

**Affiliations:** ^1^Department of Internal Medicine, Division Endocrinology and Metabolism, Seoul National University College of Medicine, Seoul National University Bundang Hospital, Seongnam, Korea

**Keywords:** new cytokines, adiponectin, FGFs, biomarker, obesity, diabetes mellitus, insulin resistance

## Abstract

Adipose tissue is known to secrete hormones actively and produces many biologically active proteins called adipocytokines. Typically, obesity is followed by low-grade inflammation, which is characterized by increased circulating levels of pro-inflammatory cytokines. Macrophages play a role in the inflammatory process by secreting many cytokines such as tumor necrosis factor alpha, interleukin-6, resistin, and retinol binding protein-4. These cytokines and chemokines participate in low-grade pro-inflammatory processes leading to insulin resistance, metabolic impairment, and cardiovascular diseases. More metabolic regulators, such as fibroblast growth factor (FGF)21, FGF19, FGF1, vaspin, and visfatin have now been discovered but their exact roles in human diseases are still unclear. This review focuses on recent research regarding the role of adipokines and new metabolic factors in metabolic derangement or cardiovascular disease.

## Introduction

The prevalence of obesity is increasing throughout the world. Obesity is associated with a broad spectrum of cardiometabolic disorders, including hypertension, dyslipidemia, diabetes, and cardiovascular disease (CVD) (1). Obesity is a heterogeneous disorder characterized by multifactorial etiology, which is characterized by various processes: changes in adipocytokines, activation of low-grade inflammation, and production of reactive oxygen species. These factors are linked to endothelial dysfunction, oxidative stress, and inflammatory processes and finally lead to the development of atherosclerosis by multiple interactive pathways (2, 3).

Adipose tissue deposition shows distinct differences between different body areas. These include anatomical, cellular, molecular, physiological, clinical, and prognostic differences. Many studies have suggested that when compared with subcutaneous adipose tissue, visceral adipose tissue and other ectopic fats are more cellular, vascular, and innervated, with a larger number of inflammatory and immune cells, less pre-adipocyte differentiation, and a greater percentage of large adipocytes (4, 5).

As adiposity increases in visceral and ectopic areas, macrophages may increase infiltration (6). This cross-talk between adipose tissue and macrophages is a source of many cytokines such as tumor necrosis factor alpha (TNFα), interleukin (IL)-6, resistin, retinol binding protein-4 (RBP4), which are suspected to participate in low-grade pro-inflammatory processes leading to metabolic disorders, insulin resistance, and CVDs (7). Other adipokines, such as visfatin and vaspin, have been discovered but their exact roles are still unknown. Emerging metabolic regulators such as fibroblast growth factor 21 (FGF21), other FGFs and myonectin appear to play roles in obesity and insulin resistance, from our experience. This review focuses on recent updates regarding the contribution of adipokines and newly discovered metabolic regulators to obesity and insulin resistance.

## Adiponectin

Adiponectin has attracted considerable attention among the many adipocytokines secreted from adipose tissue because of its insulin-sensitizing property. Early studies showed that adiponectin levels were low in patients with impaired glucose homeostasis or type 2 diabetes (8, 9). Since then, many studies have demonstrated a significant inverse association between adiponectin and insulin resistance (9). Prospective studies have proved that low levels of adiponectin are associated with an increased incidence of type 2 diabetes (10, 11). Based on this finding, intervention studies focusing on exercise have been tried. A lifestyle intervention study with overweight/obese children for 1 year increased circulating adiponectin levels and insulin sensitivity significantly (12). Our group also proved that adiponectin levels increased significantly after a 10-week aerobic training program in healthy young and middle-aged women, and this was associated with improvements in insulin sensitivity (13).

Adiponectin is closely associated with atherogenesis and the development of CVDs. Low plasma adiponectin level was a predictor of CVD outcome such as myocardial infarction in the general population and among patients with diabetes or end-stage renal disease (14–17). Interestingly, several studies suggested that the high molecular weight form of adiponectin is a more accurate independent risk factor for CVD than the whole adiponectin level (17–19). However, no significant association between adiponectin and the risk of CVD was found after adjustment for potential confounders (11).

From mechanistic studies in endothelial cells, it was proved that adiponectin strongly inhibits the production of inflammatory cytokines and adhesion molecules, including ICAM-1, VCAM-1, and E-selectin (19). Those results suggest that high levels of adiponectin play a role against the development of atherosclerosis and this has been confirmed in human studies (20, 21).

Thus, many basic and some population-based studies suggest that adiponectin might have a beneficial role in metabolic diseases and atherosclerosis, but some reports are less consistent. This might arise from differences between studies such as variations in populations, confounding factors (or lack thereof) and different isoforms of adiponectin (total vs. the high molecular weight form).

## Resistin

Initially, resistin was discovered as an adipocytokine in animal models. It was suspected to link obesity with diabetes because it was produced mainly by adipocytes (22). By contrast, adipocytes seem to contribute only a small fraction of the resistin production in humans (23). Instead, inflammatory cells such as macrophages are considered the predominant source of circulating resistin (24).

Some studies have reported that resistin levels are increased in obese individuals (25, 26) while others have not (27, 28). Population-based studies have shown that resistin levels are associated with metabolic impairments and insulin resistance (27, 29, 30) but the association between resistin levels and insulin sensitivity has been inconsistent in humans (25, 31). Resistin levels have also been associated with coronary heart diseases (32) and were correlated with calcification deposition in coronary arteries (28, 33). In contrast, other studies have not shown a significant association between resistin and coronary artery diseases (18, 34). Thus, the evidence linking resistin with decreased insulin sensitivity or increased cardiovascular risk remains inconsistent.

Of note, the secretions of TNFα, IL-6, and other cell adhesion molecules are increased by resistin (35). An *in vitro* study demonstrated that resistin treatment increased the proliferation and migration of vascular smooth muscle and endothelial cells (36). In summary, resistin may participate in cardiovascular physiopathology in humans via the action of macrophages implicated in the inflammatory response related to obesity.

## Retinol Binding Protein-4

Decades ago, RBP4 was discovered as an adipocytokine that binds specifically to vitamin A (37). RBP4 is produced mainly by the liver and adipose tissue (38). RBP4 levels are closely associated with obesity, particularly visceral adiposity in mice and humans (38, 39). Elevated RBP4 levels were associated with a clustering of components of metabolic syndrome in insulin-resistant subjects (39). In population-based studies, RBP4 levels were positively associated with the obesity index, high blood pressure, and unfavorable lipid profiles (40). RBP4 levels were increased in naive hypertensive women and were correlated with the degree of intima-media thickness, suggesting a participation of this adipocytokine in modulation of the atherosclerotic process and cardio- and cerebrovascular diseases (41, 42). Our group published data showing that regular exercise intervention with a 10-week, moderate-intensity regimen improved cardiorespiratory fitness and adipocytokines including RBP4 levels (13). Weight loss induced by bariatric surgery also decreased RBP4 concentrations (43). In addition, our group also reported that plasma RBP4 levels were significantly higher among patients converting to full diabetes mellitus (DM) from previous gestational DM compared with non-DM converters (44) and plasma RBP4 levels showed significant correlation with cardiovascular risks in patients with subclinical hypothyroidism (45). A recent study with dyslipidemia subjects found that circulating RBP4 concentrations were associated with small dense low-density lipoprotein (LDL) cholesterol and oxidized LDL levels (46).

Although there was robust evidence suggesting role of RBP4 in abnormal glucose metabolism and development of atherosclerosis in mice, several human studies reported that the serum level of RBP4 was not associated with obesity or insulin sensitivity (47, 48). Janke et al. reported discrepancy of relationship of RBP4 with glucose homeostasis between rodents and human (47). Promintzer et al. also demonstrated no increase of plasma RBP4 levels and no correlation with insulin sensitivity in insulin-resistant humans (48).

Since evidence showing relationship of RBP4 with cardiometabolic risk in human is inconsistent, there is still argument on whether elevated RBP4 levels contribute to the pathogenesis of abnormal glucose homeostasis or insulin resistance. More data are needed to clarify the potential role of RBP4 in abnormal metabolic consequences.

## C1q Tumor Necrosis Factor-α-Related Protein Isoform 5

**Figure 1:**
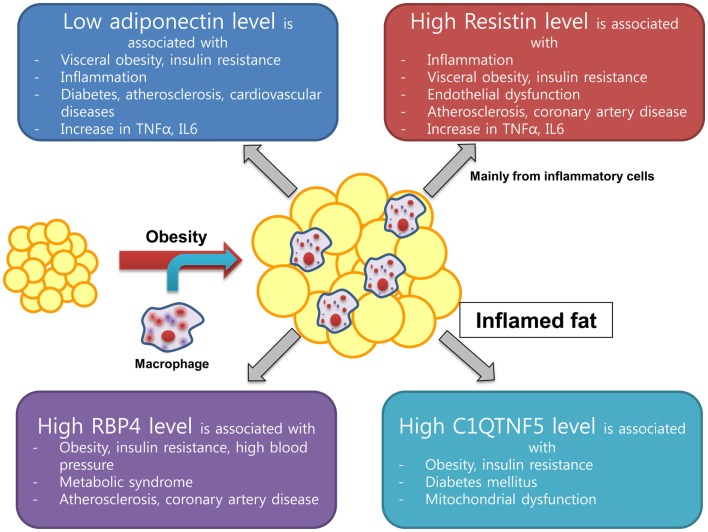
**New emerging adipokine from inflamed fat and its association with insulin resistance and cardiovascular health**.

The C1q TNF-α-related proteins or myonectins have drawn recent attention and C1q TNF-α-related protein isoform 5 (C1QTNF5) has been a focus of research because of its possible association with cardiometabolic risk (49). Structurally, C1QTNF5 is similar to adiponectin in its domain structure. C1QTNF5 belongs to a family of proteins characterized by an N-terminal signal peptide, a collagen repeat domain, and a C-terminal C1q-like globular domain (50). C1QTNFs are expressed in many tissues and have more structural or extracellular matrix-related functions than adiponectin (51). Recently, it was found that the C1QTNF5 level increased in mitochondrial (mt) DNA-depleted myocytes and this was associated with elevated adenosine monophosphate-activated protein kinase (AMPK) activity. In addition, the serum level of C1QTNF5 increased significantly in obese/diabetic animals (52). C1QTNF5 gene was upregulated from the microarray result of subcutaneous fat in obese Pima Indians, suggesting its possible role in developing obesity (53). Our group found that a 10-week exercise training program performed at moderate-intensity decreased C1QTNF5 levels and insulin resistance parameters and increased cardiorespiratory fitness, mtDNA density, and adiponectin level in both young and older groups of women (54). These findings suggest that C1QTNF5 might be an important factor linking mitochondrial dysfunction with insulin resistance. Further research is needed to identify the role and molecular mechanism of C1QTNF5 in the development of insulin resistance. Integrated schematic figure from adiponectin to C1QTNF is in Figure 1.

## Fibroblast Growth Factor 21

Fibroblast Growth Factor (FGF)21, FGF19, and FGF23 belong to the FGF19 family that acts in hormone-like manners unlike other FGF species (55). FGF21 has been highlighted as a new drug candidate for enhancing insulin sensitivity, inducing lipolysis, and preventing diet-induced obesity in many *in vitro* and *in vivo* studies (56–58). FGF21 is mainly produced in the liver but acts on adipose tissue due to its preference for binding to FGF receptor 1 (55, 59). In humans, serum FGF21 levels are paradoxically increased in metabolic diseases such as obesity, diabetes, and CVD (60–62), which infer FGF21 resistance in humans. There are several lines of evidence from animal studies to explain FGF21 resistance in the receptor and in the post-receptor signaling pathway but there is no clear mechanism in humans so far. Including the results of our group, FGF21 excretion in humans is dependent on residual renal function based on data from patients in end-stage renal disease undergoing peritoneal dialysis and hemodialysis (63, 64). We reported that serum FGF21 concentration was significantly associated with altered lipid profiles, especially with hypertriglyceridaemia, insulin resistance, metabolic syndrome, and ectopic fat deposition when adjusted for the body mass index (65). Recently, many interesting features about the role of FGF21 in metabolism have been published. FGF21 regulates PGC1-α protein levels and enhances white adipose tissue browning with upregulation of UCP1 and other thermogenic genes in a cold-exposure mouse model (66). Kim et al. reported that muscle-specific deletion of the *Atg7* (autophagy-related 7) gene in mice produced mitochondrial dysfunction and promoted FGF21 expression, showing a phenotype of being insulin-sensitive and resistant to diet-induced obesity (67). FGF21 enhanced peroxisome proliferator-activated receptor gamma (PPARγ) desumoylation in fat cells to increase its action and showed association with lower bone mass caused by PPARγ activation *in vivo* (68, 69). There are still many unknown aspects of FGF21, especially its role in human metabolism. However, clinical trials of this molecule are ongoing and the results will help explain its effect on glucose, obesity, and lipid metabolism in humans more clearly.

## Other FGFs

**Figure 2:**
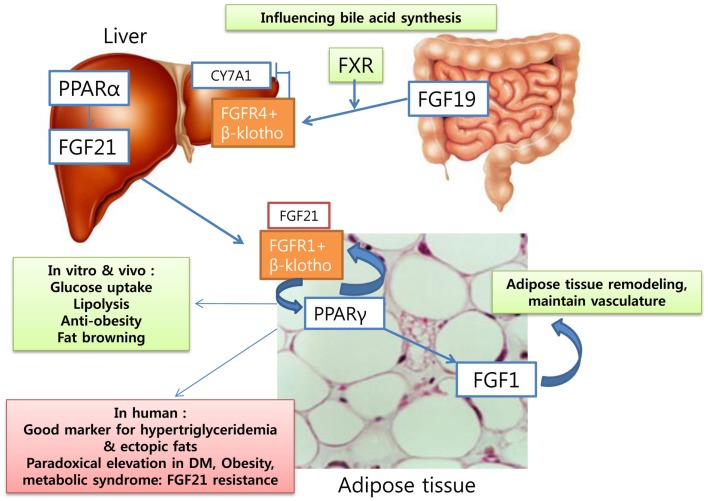
**Fibroblast growth factors: as metabolic regulators in human**.

FGF19 in humans and its mouse ortholog FGF15 have been studied for their roles in controlling bile acid synthesis. Recent data showed that activation of the farnesoid X receptor (FXR) by bile acids induced FGF19 and FGF receptor 4-mediated JNK/ERK pathways and inhibited the *CY7A1* gene encoding cholesterol 7α hydroxylase (70). In other study, transintestinal flux of bile acids with diurnal variation to control FGF19 formation in the intestine (71). The peak FGF19 formation was made 90–120 min after postprandial rise of serum bile acids. FGF19 is also a member of the FGF19 family, like FGF21 and FGF23, and acts in a hormone-like manner with a possible role in cholesterol metabolism through bile acid synthesis. In addition, FGF1 has a role in adipose tissue remodeling in mice fed a high-fat diet, being regulated by PPARγ activation. Mice lacking FGF1 showed abnormal adipose tissue with aberrant vasculature and a severe diabetic phenotype in high-fat dietary conditions (72). FGF1 is known for its role in wound healing and development (73), but it is now seen to have a role in adipose tissue remodeling and a possible link with obesity. The possible role of FGF 19 family in human is in Figure 2.

## Vaspin

Vaspin is an adipocytokine isolated from visceral adipose tissue of an animal model of abdominal obesity with type 2 diabetes (OLETF rat). It is increased in the prediabetic stage and decreases when the OLETF rats develop overt diabetes. In animals, vaspin treatment ameliorates insulin sensitivity in high-fat- or high sucrose-induced diabetes models (74) and protects against endothelial cell damage caused by free fatty acids through the PI3 kinase/Akt pathway (75). It has been suggested to be a “good” adipocytokine, such as adiponectin. However, serum vaspin levels were paradoxically elevated in human subjects with diabetes and obesity (76). We also reported that the serum vaspin level was higher in women than in men and it was correlated with the metabolic syndrome in men and coronary atherosclerosis in women. However, men with longer duration of diabetes and microvascular complications showed significantly lower levels of vaspin (77). More data are needed to understand the role of vaspin in human diseases such as atherosclerosis, diabetes, and obesity.

## Visfatin

Visfatin was first isolated from the visceral fat of humans and mice and showed insulin-like action by binding to the insulin receptor (78). In carotid artery atheromatous plaques, immunohistochemistry for visfatin showed much higher expression in unstable, symptomatic patients compared with asymptomatic patients, suggesting a role in generating macrophage foam cells in atheromata (79). The serum visfatin level was significantly reduced after gastric bypass surgery in morbidly obese subjects (80). Because visfatin is a pre B cell colony-enhancing factor, it has been studied in modulating systemic inflammation. In CD14+ monocytes, visfatin induced the expression of IL-1β, TNF-α, IL-6, and other CD molecules (81). In addition, the serum visfatin level was higher in patients with diabetes and diabetic nephropathy (82). Visfatin is now regarded as an extracellular nicotinamide phosphoribosyltransferase (eNampt) enzyme and plays an important role in insulin secretion from pancreatic β cell by systemic nicotinamide adenine dinucleotide (NAD) biosynthesis (83). *In vitro* and *in vivo*, visfatin mimics insulin action, but in human studies, it is paradoxically increased in disease conditions and shows correlation with systemic inflammation, vascular complications, and insulin secretion. However, more studies are needed to clarify the role of visfatin in humans.

## Conclusion

In summary, adipose and muscle tissues are now recognized as important and active endocrine organs. More adipocytokines and metabolic regulators are being discovered continuously and the clinical implications of these molecules are important in understanding the pathophysiology of human obesity, insulin resistance, and CVD. The “classic” adipocytokines such as adiponectin, TNF-α, and IL-6 have been regarded as consistent surrogate markers to reflect cardiovascular risk and other metabolic abnormalities in subjects with insulin resistance. Adiponectin was considered as a good biomarker to protect atherosclerosis and to reduce systemic inflammation from many studies that we mentioned in this review. However, recent studies suggested so called “adiponectin paradox” in many studies, which showed increased adiponectin levels were correlated with higher cardiovascular or all-cause mortality in epidemiological data (84–86). The underlying mechanism behind this paradox is still unclear, but we can assume that there could some compensatory elevation of adiponectin in patients with metabolic abnormalities resulted in the association with higher mortality in the future.

Some evidence provides linking resistin and RBP4 with insulin resistance or cardiovascular risk. However, there are inconsistent results suggesting no or weak relationship of these factors with obesity and insulin sensitivity. For example, RBP4 was discovered from adipose tissue specific GLUT4 knockout mouse to explain strong insulin resistance in this animal model. However, the inconsistent association with insulin resistance parameters in different clinical settings of various studies which included patients with obesity, DM, different ethnicity, and CVD, it could not convince us to believe the role of RBP4 would be a causality of insulin resistance in metabolic diseases.

As for the emerging metabolic regulators such as FGFs (FGF21, FGF19, and FGF1), adipokines from visceral fat (vaspin and visfatin) and myokines, we need more studies to clarify their role in human diseases. FGF21 has been developed as a new drug for antidiabetic medication and human trial is on-going based on its favorable results from *in vitro* and *in vivo*. We have to wait and see the result of human trials that we could fully understand the mechanism of antidiabetic or any metabolic effects on human body. The possible role of FGF21 through PPAR α and γ in the liver and in fat tissues can be expected to have beneficial effects on insulin signaling pathway to ameliorate metabolic abnormalities. It is still a question whether these new metabolic regulators are just surrogate markers or might be causes of obesity and insulin resistance.

From this perspective, it is too early yet to apply these adipocytokines or metabolic regulators as predictors for cardiometabolic risk in the clinical practice. Up to date, it could be used as a surrogate biomarker to reflect metabolic abnormalities in many metabolic diseases. More mechanistic experiments and long-term outcome studies are warranted to elucidate the active role of these factors in the physiopathology of cardiometabolic disorders to identify their clinical implications at bedside level.

## Conflict of Interest Statement

The authors declare that the research was conducted in the absence of any commercial or financial relationships that could be construed as a potential conflict of interest.
